# Validation of MELD3.0 in 2 centers from different continents

**DOI:** 10.1097/HC9.0000000000000504

**Published:** 2024-07-31

**Authors:** Marta Tejedor, José María Bellón, Margarita Fernández de la Varga, Peregrina Peralta, Eva Montalvá, Nazia Selzner, Marina Berenguer

**Affiliations:** 1 Hepatology, Hospital Universitario Infanta Elena, Valdemoro, Madrid, Spain; 2 Biostatistics, Instituto de Investigación Sanitaria Gregorio Marañón (IISGM), Madrid, Spain; 3 CIBERINFEC, ISCIII—CIBER de Enfermedades Infecciosas, Instituto de Salud Carlos III, Madrid, Spain; 4 Hepatology, Hospital Universitario y Politécnico La Fe, Valencia, Spain; 5 Research, Toronto General Hospital, Toronto, Ontario, Canada; 6 Department of Hepatobiliopancreatic Surgery and Transplantation Unit, Hospital Universitario y Politécnico La Fe, Valencia, Spain; 7 Department of Medicine, University of Valencia, Valencia, Spain; 8 Liver and Digestive Diseases Networking Biomedical Research Centre (CIBERehd), Instituto de Salud Carlos III, Madrid, Spain; 9 Instituto de Investigación Sanitaria (IIS) La Fe, Valencia, Spain; 10 Transplant Hepatology, Ajmera Transplant Center, University of Toronto, Toronto, Ontario, Canada; 11 Hepatology and Liver Transplant Unit, Hospital Universitario y Politécnico La Fe, Valencia, Spain; 12 Medicine Department, University of Valencia, Valencia, Spain

## Abstract

**Background:**

MELD3.0 has been proposed to stratify patients on the liver transplant waiting list (WL) to reduce the historical disadvantage of women in accessing liver transplant. Our aim was to validate MELD3.0 in 2 unique populations.

**Methods:**

This study is a 2-center retrospective cohort study from Toronto, Canada, and Valencia, Spain, of all adults added to the liver transplant WL between 2015 and 2019. Listing indications whose short-term survival outcome is not adequately captured by the MELD score were excluded. All patients analyzed had a minimum follow-up of 3 months after inclusion in the WL.

**Results:**

Six hundred nineteen patients were included; 61% were male, with a mean age of 56 years. Mean MELD at inclusion was 18.00 ± 6.88, Model for End-Stage Liver Disease Sodium (MELDNa) 19.78 ± 7.00, and MELD3.0 20.25 ± 7.22. AUC to predict 90-day mortality on the WL was 0.879 (95% CI: 0.820, 0.939) for MELD, 0.921 (95% CI: 0.876, 0.967) for MELDNa, and 0.930 (95% CI: 0.888, 0.973) for MELD3.0. MELDNa and MELD3.0 were better predictors than MELD (*p* = 0.055 and *p* = 0.024, respectively), but MELD3.0 was not statistically superior to MELDNa (*p* = 0.144). The same was true when stratified by sex, although the difference between MELD3.0 and MELD was only significant for women (*p* = 0.032), while no statistical significance was found in either sex when compared with MELDNa. In women, AUC was 0.835 (95% CI: 0.744, 0.926) for MELD, 0.873 (95% CI: 0.785, 0.961) for MELDNa, and 0.886 (95% CI: 0.803, 0.970) for MELD3.0; differences for the comparison between AUC in women versus men for all 3 scores were nonsignificant. Compared to MELD, MELD3.0 was able to reclassify 146 patients (24%), the majority of whom belonged to the MELD 10–19 interval. Compared to MELDNa, it reclassified 68 patients (11%), most of them in the MELDNa 20–29 category.

**Conclusions:**

MELD3.0 has been validated in centers with significant heterogeneity and offers the highest mortality prediction for women on the WL without disadvantaging men. However, in these cohorts, it was not superior to MELDNa.

## Graphical abstract

**Figure:**
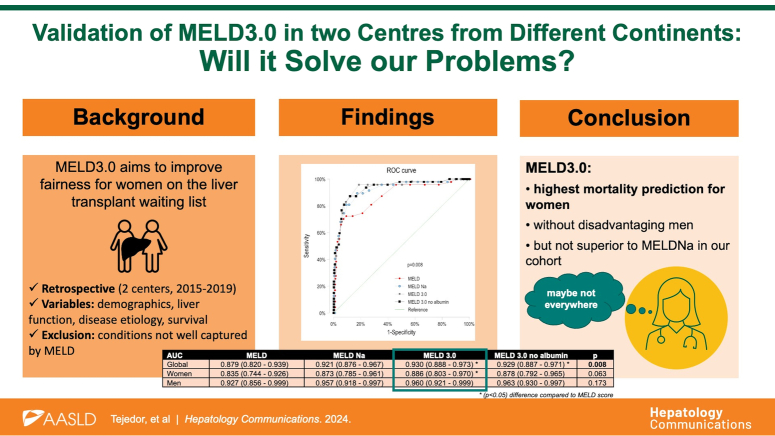

## INTRODUCTION

Liver transplant (LT) is the treatment of choice in end-stage liver disease, but the scarcity of donor organs for the increasing pool of potential recipients limits the access of patients to this life-saving procedure. In the majority of Western countries, allocation of organs on the waiting list (WL) is based on an urgency model (Model for End-Stage Liver Disease [MELD] and Model for End-Stage Liver Disease Sodium [MELDNa]), which prioritizes the sickest patients.^1^

Several studies based on large data sets show that women are disadvantaged in accessing LT, with a 30% increased odds of death and a 10% higher risk of delisting for becoming too sick for LT compared to men.^2^,^3^,^4^ In addition, the MELD allocation system is associated with a further reduction in rates of transplantation among women compared with the previous era (reduction compared with men by 9% in the pre-MELD era versus 14% in the MELD era, *p* < 0.05).^5^

To correct for the long-standing underprioritization of female WL candidates, Kim et al^6^ published, in 2021, a study on the new score MELD3.0. This new version of the MELD score updates creatinine, International Normalized Ratio, bilirubin, and sodium coefficients and incorporates sex and albumin. A version without albumin has also been developed. MELD3.0 improves the WL mortality prediction of the entire population compared to MELDNa, but in particular, it correctly reclassifies more women (15%) than men (4%). MELD3.0 has been implemented as the allocation policy in LT in the United States in July 2023.^7^

However, MELD3.0 has been developed and validated using the Organ Procurement and Transplantation Network Standard Transplant Analysis and Research (OPTN STAR) files and is thus reliant on the specific demographics and characteristics of the American WL population.^6^,^8^ Therefore, there is a need to assess its accuracy in other countries whose WL population might have different baseline characteristics.

The aim of our study was to assess MELD3.0’s performance in predicting 90-day WL mortality compared to MELD and MELDNa in 2 centers from different continents.

## METHODS

### Patients and data

In this retrospective study, the medical records of all adult patients (≥18 y old) listed for LT between January 1, 2015, and December 31, 2019, at Toronto General Hospital (Toronto, Canada) and Hospital Universitario y PolitécnicoLa Fe (Valencia, Spain) were reviewed, and data were collected until 1 year after transplant whether alive, dead or re-transplanted.

Variables related to patient’s demographics, liver function at the time of inclusion in the WL, and need for ICU, pressors, dialysis, or intubation at the time of LT were collected. Etiology of liver disease and survival, both on the WL and after LT (up to 1 year after LT), were also collected.

Indications whose short-term survival outcome is not adequately captured by the MELD score were excluded, such as fulminant liver failure without underlying liver cirrhosis, any other reason without underlying liver cirrhosis, HCC, combined organ transplant, and retransplantation.

MELD was adopted in the United States as the preferred allocation system in 2002 and MELDNa in 2016. MELD was the standard allocation system in Valencia and Toronto in 2015. From 2016 onward, each center adopted MELDNa as the standard of care. To take these differences into account, all analyses have been performed using both MELD and MELDNa as the reference category.

### Data analysis

IBM SPSS Statistics for Windows, Version 25.0 (IBM Corp.) and Stata 15 were used for all calculations. Categorical variables were expressed as frequencies and percentages and compared with the chi-square or Fisher exact tests. Continuous variables were expressed as mean (SD) or median (percentile 25; percentile 75) and compared using the Student *t* test or the Mann-Whitney test in case of non-normal distributions. *p* values <0.05 were considered statistically significant.

Overall survival of the 2 cohorts was calculated from inclusion on the WL until death or last visit. Survival curves were constructed with the Kaplan-Meier method and compared with the log-rank test. Multivariate Cox regression analysis was performed, including independent variables such as transplant, cohort of origin, age, sex, MELD3.0 ≥30, and PVT. These variables were selected following clinical criteria. Transplant status was considered a time-dependent variable because of its significant influence on the probability of death. This decision is based on the fact that transplantation substantially impacts the time course of patient survival, altering their risk of events over the course of follow-up. The ability of MELD scores to predict mortality in the first 90 days after inclusion in the WL was studied with the area under the ROC curve (receiver operating characteristic curves; AUC) and the Harrell c-statistic, considering transplantation as a competing event. The AUC allows the predictive ability of mortality scores to be measured as if it were a 90-day cross-sectional study, while the Harrell c-statistic takes into account the time of death within the 90-day interval. Both statistics are interpreted in a similar way, with 1 being the maximum possible value indicating perfect prediction. These analyses were performed using 2 different approaches: including or excluding patients transplanted in the first 90 days.

This study is compliant with ethical guidelines and the Declaration of Helsinki and has gained the Research Ethics Board approval (number 19-5798) at the UHN Toronto General Hospital. A waiver of consent was obtained from the board in view of the retrospective nature of the data collected.

## RESULTS

### Baseline characteristics of patients on the WL

A total of 619 patients have been included and analyzed, 448 from the Toronto site and 171 from the Valencia site. Sixty-one percent of them were male; the mean age was 55.6 ± 10.2 years. Overall, the main indication for LT was alcohol-associated liver disease (41%). The baseline demographics of both cohorts can be found in Table 1. In summary, Canadian patients were younger, taller, and sicker at the time of transplant (a higher proportion of them needing pressors or intubation at the time of transplant) despite similar MELD scores at listing. The etiology of liver disease was different between the 2 cohorts, with a predominance of alcohol and virus-related liver disease in the European one and metabolically associated steatotic liver disease in the Canadian cohort.

**TABLE 1:** Baseline characteristics of both cohorts

Variable	Valencia cohort (n = 171)	Toronto cohort (n = 448)	*p*
Age (y)	57.51 ± 9.40	54.88 ± 10.39	**0.004**
Height (cm)	167.80 ± 8.26	169.40 ± 9.90	0.053
Weight (kg)	77.33 ± 17.62	79.25 ± 20.78	0.286
ICU^a^, n (%)	4 (2.9)	8 (3.5)	1.000
Pressors^a^, n (%)	1 (0.7)	10 (4.4)	0.057
Intubation^a^, n (%)	0 (0.0)	12 (5.3)	**0.004**
HD^a^, n (%)	3 (2.2)	12 (5.3)	0.179
MELD^b^	17.56 ± 5.66	18.16 ± 7.29	0.337
MELDNa^b^	18.83 ± 6.04	20.13 ± 7.30	**0.041**
MELD3.0^b^	19.16 ± 6.09	20.66 ± 7.56	**0.022**
MELD3.0 no albumin^b^	19.43 ± 6.24	20.68 ± 7.75	0.062
Etiology, n (%)			
ALD	96 (56.1)	158 (35.3)	**<0.001**
Viral	70 (40.9)	84 (18.7)	**<0.001**
MASLD	12 (7.0)	110 (24.6)	**<0.001**
AIH	6 (3.5)	32 (7.1)	0.132
PSC	7 (4.1)	66 (14.7)	**<0.001**
PBC	12 (7.0)	40 (8.9)	0.519
Others	11 (6.5)	71 (15.8)	**0.002**

*Note*: Results of quantitative variables expressed as mean ± SD. Categorical variables are expressed as n (%).Welch 2-sample *t* test for comparison between both cohorts (quantitative variables). Pearson chi-squared or Fisher exact test (categorical variables).

Bold values indicate statistical significance.

^a^ At the time of transplantation.

^b^ At the time of listing.

Abbreviations: AIH, autoimmune hepatitis; ALD, alcohol-associated liver disease; HD, hemodialysis; ICU, intensive care unit; MASLD, metabolic dysfunction–associated steatotic liver disease; MELD, Model for End-Stage Liver Disease; MELDNa, Model for End-Stage Liver Disease Sodium; PBC, primary biliary cholangitis; PSC, primary sclerosing cholangitis.

### Comparison of MELD, MELDNa, and MELD3.0 for mortality prediction

**FIGURE 1:**
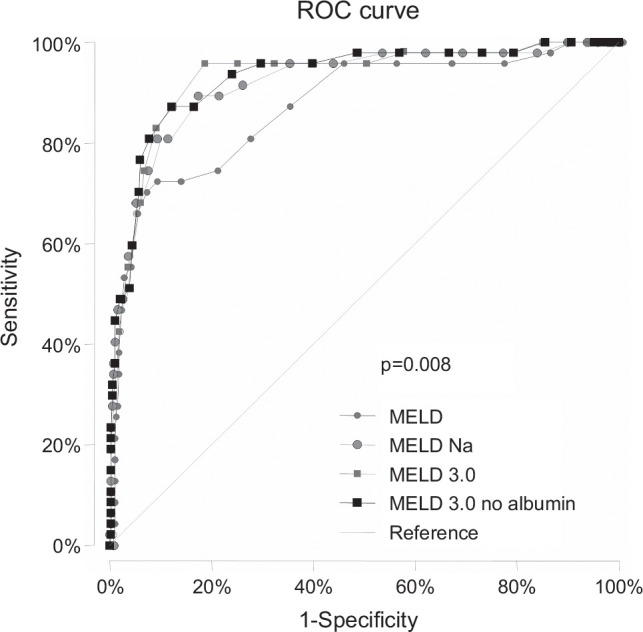
Comparison of areas under the curve for 90-day mortality prediction on the waiting list between the different scoring systems. MELD3.0 offered the best prediction (*p* = 0.008). Transplanted patients in the first 90 days are excluded. Abbreviations: MELD, Model for End-Stage Liver Disease; ROC, receiver operating characteristic.

An initial AUC analysis excluding patients transplanted in the first 90 days was performed. MELD3.0 offered the best 90-day WL mortality prediction compared to the other scoring systems (Figure 1) and performed statistically better than MELD (*p* = 0.024) but not than MELDNa (*p* = 0.14). The same was true when stratified by sex, although the difference between MELD3.0 and MELD was only significant for women (*p* = 0.032), while no statistical significance was found in either sex when compared with MELDNa (Table 2). No statistical differences were found when comparing each scoring system between women and men (Table 2). Similar results were obtained when a time-dependent approach (Harrell c-statistic) was used. In this case, when transplanted patients were included, transplantation was considered a competing event (Table 2).

**TABLE 2:** Comparison of the different scoring systems for the prediction of 90-day mortality on the waiting list in the global cohort and stratified by sex

	MELD	MELDNa	MELD3.0	MELD3.0 no albumin	*p*
AUC^a^					
Global (n = 435)	0.879 (0.820–0.939)	0.921 (0.876–0.967)	0.930 (0.888–0.973)*	0.929 (0.887–0.971)*	**0.008**
Women (n = 176)	0.835 (0.744–0.926)	0.873 (0.785–0.961)	0.886 (0.803–0.970)*	0.878 (0.792–0.965)	0.063
Men (n = 259)	0.927 (0.856–0.999)	0.957 (0.918–0.997)	0.960 (0.921–0.999)	0.963 (0.930–0.997)	0.173
AUC^b^					
Global (n = 615)	0.812 (0.747–0.877)	0.853 (0.802–0.905)	0.856 (0.812–0.908)	0.858 (0.809–0.907)*	**0.046**
Women (n = 240)	0.747 (0.646–0.848)	0.795 (0.702–0.889)	0.800 (0.712–0.888)	0.792 (0.699–0.884)	0.172
Men (n = 375)	0.874 (0.797–0.950)#	0.898 (0.846–0.950)	0.901 (0.851–0.951)	0.903 (0.856–0.951)#	0.340
Harrell c^a^					
Global (n = 435)	0.868 (0.811–0.926)	0.903 (0.856–0.951)	0.913 (0.869–0.957)*	0.910 (0.867–0.954)*	**0.018**
Women (n = 176)	0.826 (0.728–0.923)	0.852 (0.755–0.949)	0.868 (0.775–0.961)*	0.856 (0.759–0.952)	0.059
Men (n = 259)	0.909 (0.842–0.976)	0.939 (0.899–0.979)	0.941 (0.902–0.981)	0.945 (0.911–0.978)#	0.169
Harrell c^c^					
Global (n = 615)	0.816 (0.752–0.881)	0.844 (0.790–0.899)	0.856 (0.805–0.906)*	0.849 (0.798–0.901)*	0.106
Women (n = 240)	0.763 (0.654–0.871)	0.785 (0.678–0.891)	0.799 (0.699–0.899)	0.784 (0.678–0.890)	0.143
Men (n = 375)	0.861 (0.786–0.936)	0.886 (0.835–0.938)	0.892 (0.843–0.942)	0.892 (0.845–0.939)#	0.338

* (*p* < 0.05) difference compared to MELD score.

# (*p* < 0.05) difference comparing by sex.

Bold values indicate statistical significance.

^a^ Transplant patients are excluded.

^b^ Transplant patients are included.

^c^ Transplantation is considered a competing event.

Abbreviations: MELD, Model for End-Stage Liver Disease; MELDNa, Model for End-Stage Liver Disease Sodium.

The performance of the different scoring systems in each of the cohorts separately is presented in Supplemental Figure S1, http://links.lww.com/HC9/A1000. When the analysis was repeated including all patients in both cohorts, both transplanted and not in the first 90 days, the results were similar (Supplemental Figure S2, http://links.lww.com/HC9/B2).

**FIGURE 2:**
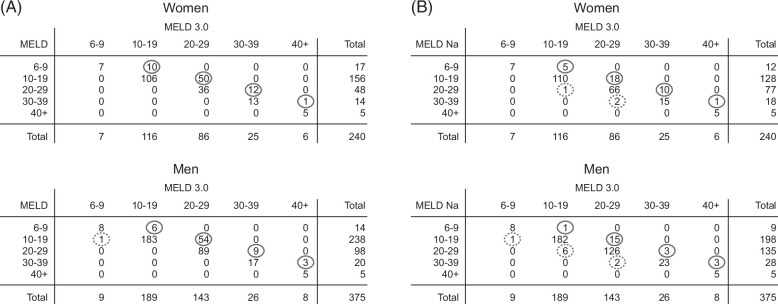
Number of patients reclassified by MELD3.0 by sex. (A) Compared to MELD. Overall. Thirty percent of women were reclassified as gaining priority when using MELD3.0 versus MELD as opposed to 19% for men (*p* = 0.002). (B) Compared to MELDNa. Overall. Fourteen percent of women were reclassified as gaining priority when using MELD3.0 versus MELDNa as opposed to 6% for men (*p* < 0.001). Red circles indicate the number of patients who gain priority. Blue dotted circle indicates the number of patients who lose priority. The main diagonal indicates patients who do not change priority. Abbreviations: MELD, Model for End-Stage Liver Disease; MELDNa, Model for End-Stage Liver Disease Sodium.

All included patients, whether transplanted or not in the first 90 days, were analyzed to assess MELD3.0’s reclassification ability. Compared to MELD, MELD3.0 was able to reclassify 146 patients (24%) overall, 145 of them gaining priority and 1 losing it. The majority of the reclassified patients belonged to the MELD 10–19 interval. Overall, 30% of women (n = 73) were reclassified, gaining priority when using MELD3.0 versus MELD as opposed to 19% for men (n = 72) (*p* = 0.002). None of the female patients lost priority compared to 1 male (Figure 2A). Compared to MELDNa, MELD3.0 reclassified 68 patients (11%) in total, 56 of them gaining priority. Most of the reclassified patients belonged to the MELDNa 20–29 category. Fourteen percent of women (n = 34) gained priority compared to 6% of men (n = 22) (*p* < 0.001) (Figure 2B).

Differences between patients who received a transplant in the first 90 days after inclusion on the WL and those who did not can be found in Supplemental Table S1, http://links.lww.com/HC9/B3.

### Survival analysis

Overall mortality, including all patients, was greater in the Toronto cohort (HR: 1.64, 95% CI: 1.08 –2.49). When transplant was considered a time-dependent variable, no differences were observed in mortality between centers. Age (HR: 1.05, 95% CI: 1.03–1.07), baseline MELD3.0 ≥30 (HR: 4.53, 95% CI: 2.75–7.45), and not receiving a transplant (HR: 4.76, 95% CI: 2.86–7.93) were independent predictors of mortality in the multivariate analysis (Supplemental Table S2, http://links.lww.com/HC9/B4).

**FIGURE 3:**
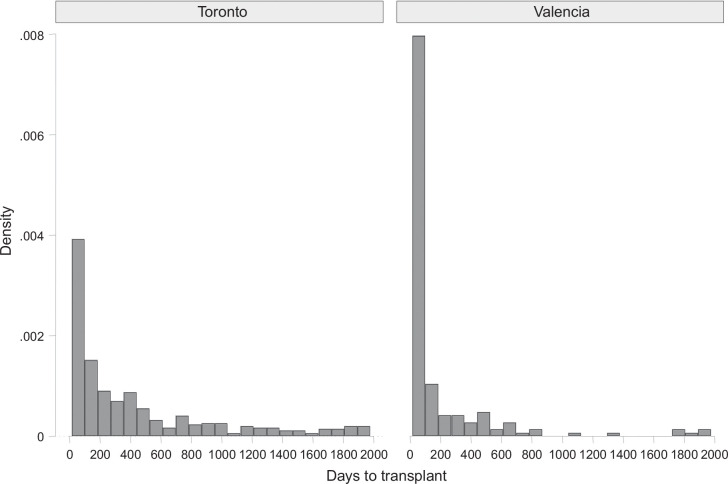
Probability density distribution of transplants for patients on the waiting list per center (*p* < 0.001).

Of note, the waiting time from listing to LT in Toronto was significantly longer (274 days [p25: 91; p75: 943] vs. 31 days [p25: 15; p75: 153], *p* < 0.001] (Figure 3).

No differences in posttransplant survival were found between cohorts.

**FIGURE 4:**
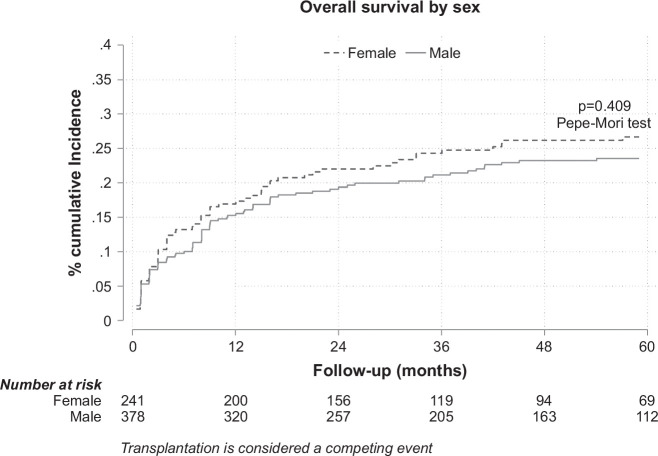
Overall survival by sex, including the pretransplant and posttransplant periods.

There were no differences in overall survival by sex (Figure 4).

## DISCUSSION

In our study, MELD3.0 offered the best 90-day WL mortality prediction compared to MELD and MELDNa and had the highest AUC and the Harrel c-statistic for women without disadvantaging men. It has thus shown good results in 2 very different study cohorts from 2 different continents. However, MELD3.0 was not superior to MELDNa in the studied sample.

Although it could seem that the WL mortality in Toronto was greater, when the multivariate analysis was performed considering LT as a time-dependent variable, there were no differences between the 2 centers. Time from listing to LT is essential, and Valencia’s waiting times are much shorter, which has a direct impact on mortality. It seems, therefore, that implementing national policies directed at reducing overall waiting times is relevant to reducing WL mortality. The “Spanish Model in Organ Donation and Transplantation” has been well described in the literature.^9^,^10^ Over the last years, the implementation of innovative measures such as the standardization of intensive care to facilitate organ donation, the expansion of donor eligibility criteria, and the incorporation of donation after circulatory death (with the systematic use of normothermic regional perfusion) has further allowed to increase the availability of livers for clinical use.^11^

The longer waiting times in Toronto could allow for further deterioration of patients on the WL, as suggested by the larger proportion of them requiring pressors or intubation at the time of transplant, despite similar MELD scores at listing compared with the Valencia cohort. This finding further supports the concept that MELD does not adequately capture all factors involved in WL mortality.^1^ Both MELDNa and MELD3.0 were better mortality predictors than MELD (*p* = 0.055 and *p* = 0.024, respectively). While MELD3.0 and MELDNa’s accuracy did not differ significantly in our study population, the AUC and the Harrel c-statistic were numerically higher for MELD3.0, which suggests that MELD3.0 could more adequately capture the severity of liver disease. In fact, its higher accuracy likely reflects the way it was constructed: for low MELD scores, hyponatremia is the most relevant variable, whereas hypoalbuminemia and female sex gain weight for medium MELD scores. Finally, as creatinine increases, albumin becomes less important, mainly for the highest MELD scores.^6^

A recent Asian study^12^ found that MELD3.0 reclassified 23% of cases to higher MELD score categories without differences in the reclassification rate between males and females. In our study, MELD3.0 predominantly reclassified women with intermediate MELD scores. Mathur et al^5^ showed that this subgroup of patients is especially disadvantaged by MELD. It remains an open question whether the use of sex as a variable itself will ultimately reduce the inequalities in access to LT of women. The main reasons proposed for the disadvantage of women in LT are the use of creatinine in the severity scoring systems, as it underestimates renal dysfunction in females due to their lower muscle mass^13^,^14^ and their smaller stature, which limits the donor pool.^15^,^16^,^17^ Therefore, the use of sex per se while keeping creatinine as a variable may not completely account for the underlying inequity reasons. In the study by Cholongitas et al,^18^ the MELD score correction by renal function accounted for a 2–3 MELD points increase in 65% of female candidates for LT, but the implementation of estimated glomerular filtration rate did not improve outcome predictions of MELD in the WL.^13^ An alternative model, GEMA, has been developed, where creatinine in the MELD formula was replaced by the Royal Free Hospital glomerular filtration rate (RFH-GFR).^19^,^20^ Patients prioritized by GEMA were more often women, in particular those at higher risk of death or delisting due to clinical deterioration.MELD3.0 does not take into account the main cause of women’s disadvantage in accessing LT, namely the underestimation of kidney dysfunction. Therefore, all women, regardless of the severity of their liver disease, receive additional points on the basis of their sex. As long as a good estimator of renal function in cirrhosis is not used instead of creatinine, it is likely that all patients with small muscle mass will be disadvantaged by the MELD-derived models compared to men. Our results also suggest that MELD3.0’s 90-day mortality prediction is better in men (numerically greater AUC), although our study did not have enough power to demonstrate statistical significance.

We initially performed an analysis excluding the transplanted patients within the first 90 days after inclusion on the WL, as they would have a greater survival probability and could bias the mortality prediction of the scores being studied. This affected mainly the Valencia cohort (n = 51 patients transplanted in this timeframe). Subsequently, we repeated the mortality prediction analysis including all the patients in both cohorts, whether transplanted or not, and found similar results, although, as expected, the AUC and Harrel c-statistic values were slightly lower for all the scores.

There has been some debate as to MELD’s short-term mortality prediction ability in recent years (c-statistic 0.80 in 2003 vs. 0.70 in 2015).^21^,^22^,^23^ Some authors, although, have argued that the c-statistic was calculated using a binary method for 90-day mortality without inputs for contributed follow-up time. When a modified Harrell c-statistic (which accounts for censoring) was used, the discriminative power of MELD was found to increase to 0.84, not significantly different from 2002.^24^,^25^ We chose to consider transplant status as a time-dependent variable because of its significant influence on the probability of death: patients who receive a transplant are more likely to survive than those who do not. However, we also performed a Cox regression model to obtain the c-Harrell statistic to ensure time (*when* the event takes place) was not influencing our results. No significant differences were shown with this approach.

Limitations of our study include the very short waiting times in the Valencia cohort and the fact that GEMA could not be calculated for comparison. Whether the very different waiting times between both cohorts play a role in the accuracy of MELD3.0 should be further explored. Given the small AUC differences between MELD3.0 and MELDNa, it is possible that our sample size did not allow us to find statistical differences between them, increasing the risk of type II error, although numerically MELD3.0’s AUC and Harrell c-statistic was higher than MELDNa.

In summary, MELD3.0 has been validated in 2 very different patient settings outside the United States. It offers the best WL mortality prediction for women without disadvantaging men, although in our cohort, it was not superior to MELDNa, which could limit its advantages, at least in certain transplant settings.

## Supplementary Material

SUPPLEMENTARY MATERIAL

## References

[1] Tejedor M Selzner N Berenguer M. Are MELD and MELDNa still reliable tools to predict mortality on the liver transplant waiting list? Transplantation. 2022;106:2122–2136. 35594480 10.1097/TP.0000000000004163

[2] Moylan CA Brady CW Johnson JL Smith AD Tuttle-Newhall JE Muir AJ. Disparities in liver transplantation before and after introduction of the MELD score. JAMA J Am Med Assoc. 2008;300:2371–2378. 10.1001/jama.2008.720 PMC3640479 19033587

[3] Cullaro G Sarkar M Lai JC. Sex-based disparities in delisting for being “too sick” for liver transplantation. Am J Transplant. 2018;18:1214–1219. 29194969 10.1111/ajt.14608 PMC5910224

[4] Mazumder NR Celaj S Atiemo K Daud A Jackson KL Kho A. Liver-related mortality is similar among men and women with cirrhosis. J Hepatol. 2020;73:1072–1081. 32344052 10.1016/j.jhep.2020.04.022 PMC7572539

[5] Mathur AK Schaubel DE Gong Q Guidinger MK Merion RM. Sex-based disparities in liver transplant rates in the United States. Am J Transplant. 2011;11:1435–1443. 21718440 10.1111/j.1600-6143.2011.03498.x PMC3132137

[6] Kim WR Mannalithara A Heimbach JK Kamath PS Asrani SK Biggins SW. MELD 3.0: The Model for End-Stage Liver Disease updated for the modern era. Gastroenterology. 2021;161:1887–1895.e4. 34481845 10.1053/j.gastro.2021.08.050 PMC8608337

[7] MELD calculator - OPTN. Accessed August 12, 2023. https://optn.transplant.hrsa.gov/data/allocation-calculators/meld-calculator/.

[8] OPTN/SRTR 2019 annual data report: Liver. Accessed October 12, 2021. Liver (hrsa.gov).

[9] Matesanz R Domínguez-Gil B Coll E de la Rosa G Marazuela R. Spanish experience as a leading country: what kind of measures were taken? Transpl Int. 2011;24:333–343. 21210863 10.1111/j.1432-2277.2010.01204.x

[10] Streit S Johnston-Webber C Mah J Prionas A Wharton G Casanova D. Ten lessons from the Spanish model of organ donation and transplantation. Transpl Int. 2023;36:11009. 37305337 10.3389/ti.2023.11009 PMC10249502

[11] Matesanz R Domínguez-Gil B Coll E Mahíllo B Marazuela R. How Spain reached 40 deceased organ donors per million population. Am J Transplant. 2017;17:1447–1454. 28066980 10.1111/ajt.14104

[12] Yoo JJ Chang JI Moon JE Sinn DH Kim SG Kim YS. Validation of MELD 3.0 scoring system in East Asian patients with cirrhosis awaiting liver transplantation. Liver Transpl. 2023;29:1029–1040. 36929833 10.1097/LVT.0000000000000126

[13] Myers RP Shaheen AAM Aspinall AI Quinn RR Burak KW. Gender, renal function, and outcomes on the liver transplant waiting list: Assessment of revised MELD including estimated glomerular filtration rate. J Hepatol. 2011;54:462–470. 21109324 10.1016/j.jhep.2010.07.015

[14] Allen AM Heimbach JK Larson JJ Mara KC Kim WR Kamath PS. Reduced access to liver transplantation in women: Role of height, MELD exception scores and renal function underestimation. Transplantation. 2018;102:1710–1716. 29620614 10.1097/TP.0000000000002196 PMC6153066

[15] Lai JC Terrault NA Vittinghoff E Biggins SW. Height contributes to the gender difference in wait-list mortality under the MELD-based liver allocation system. Am J Transplant. 2010;10:2658–2664. 21087414 10.1111/j.1600-6143.2010.03326.x PMC3059496

[16] Nephew LD Goldberg DS Lewis JD Abt P Bryan M Forde KA. Exception points and body size contribute to gender disparity in liver transplantation. Clin Gastroenterol Hepatol. 2017;15:1286–1293.e2. 28288834 10.1016/j.cgh.2017.02.033 PMC10423635

[17] Mindikoglu AL Emre SH Magder LS. Impact of estimated liver volume and liver weight on gender disparity in liver transplantation. Liver Transpl. 2013;19:89–95. 23008117 10.1002/lt.23553 PMC3535518

[18] Cholongitas E Marelli L Kerry A Goodier DW Nair D Thomas M. Female liver transplant recipients with the same GFR as male recipients have lower MELD scores—A systematic bias. Am J Transplant. 2007;7:685–692. 17217437 10.1111/j.1600-6143.2007.01666.x

[19] Rodríguez-Perálvarez ML Gómez-Orellana AM Majumdar A Bailey M McCaughan GW Gow P. Development and validation of the Gender-Equity Model for Liver Allocation (GEMA) to prioritise candidates for liver transplantation: A cohort study. Lancet Gastroenterol Hepatol. 2023;8:242–252. 36528041 10.1016/S2468-1253(22)00354-5

[20] Kalafateli M Wickham F Burniston M Cholongitas E Theocharidou E Garcovich M. Development and validation of a mathematical equation to estimate glomerular filtration rate in cirrhosis: The Royal Free Hospital cirrhosis glomerular filtration rate. Hepatology. 2017;65:582–591. 27779785 10.1002/hep.28891

[21] Godfrey EL Malik TH Lai JC Mindikoglu AL Galván NTN Cotton RT. The decreasing predictive power of MELD in an era of changing etiology of liver disease. Am J Transplant. 2019;19:3299–3307. 31394020 10.1111/ajt.15559

[22] Asrani SK Jennings LW Kim WR Kamath PS Levitsky J Nadim MK. MELD-GRAIL-Na: Glomerular filtration rate and mortality on liver-transplant waiting list. Hepatology Baltim Md. 2020;71:1766–1774. 10.1002/hep.30932 31523825

[23] Goudsmit BFJ Putter H Tushuizen ME de Boer J Vogelaar S Alwayn IPJ. Validation of the Model for End‐stage Liver Disease sodium (MELD‐Na) score in the Eurotransplant region. Am J Transplant. 2021;21:229–240. 32529758 10.1111/ajt.16142 PMC7818465

[24] Kwong A Mannalithara A Kim WR. Reply to: “The decreasing predictive power of MELD in an era of changing etiology of liver disease. Am J Transplant. 2020;20:901–902. 31814299 10.1111/ajt.15733

[25] D’Amico G Maruzzelli L. MELD calibration. Am J Transplant. 2021;21:438–439. 32786172 10.1111/ajt.16255

