# Supplementary material for: Validation of MELD3.0 in 2 centers from different continents
Source: Hepatol Commun. 2024 Jul 31;8(8):e0504. doi: 10.1097/HC9.0000000000000504 (PMC12333758; doi:10.1097/HC9.0000000000000504)
Supplement: SUPPLEMENTARY MATERIAL [file hc9-8-e0504-s001.docx]

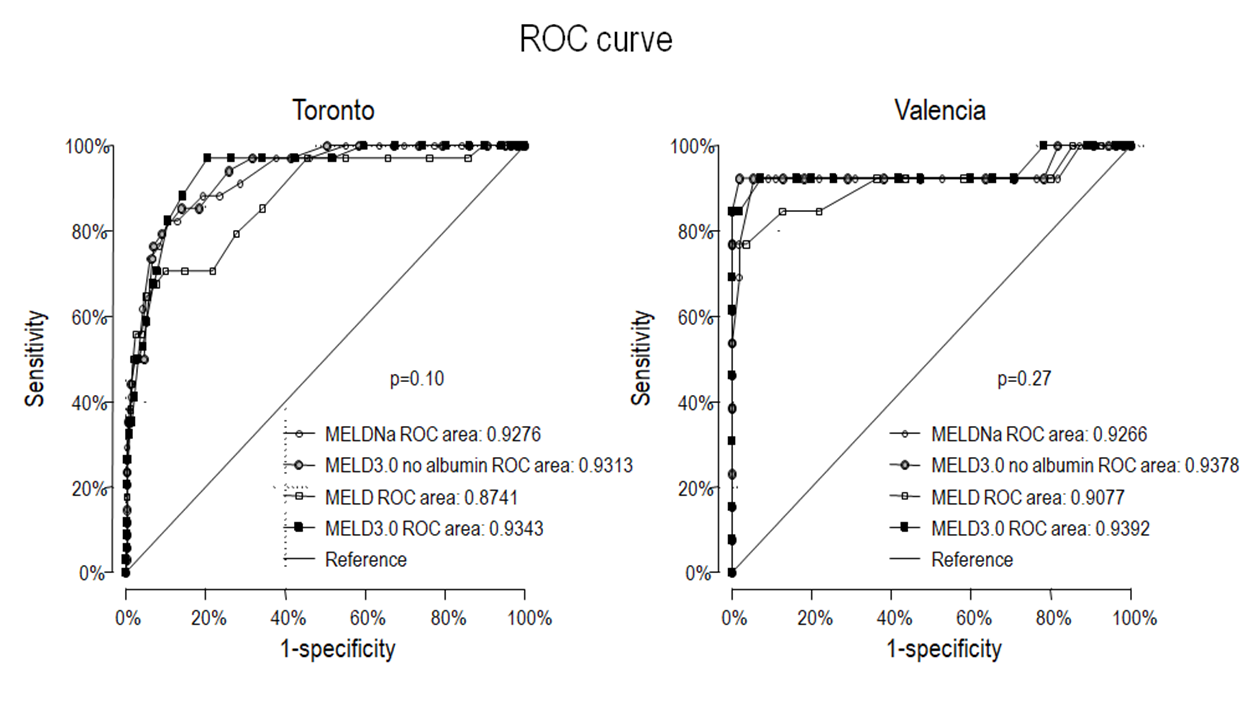


Supplementary Figure 1. Performance of the different scoring systems in each of the separate cohorts.
