# Supplementary material for: Validation of MELD3.0 in 2 centers from different continents
Source: Hepatol Commun. 2024 Jul 31;8(8):e0504. doi: 10.1097/HC9.0000000000000504 (PMC12333758; doi:10.1097/HC9.0000000000000504)
Supplement: SUPPLEMENTARY MATERIAL [file hc9-8-e0504-s002.docx]

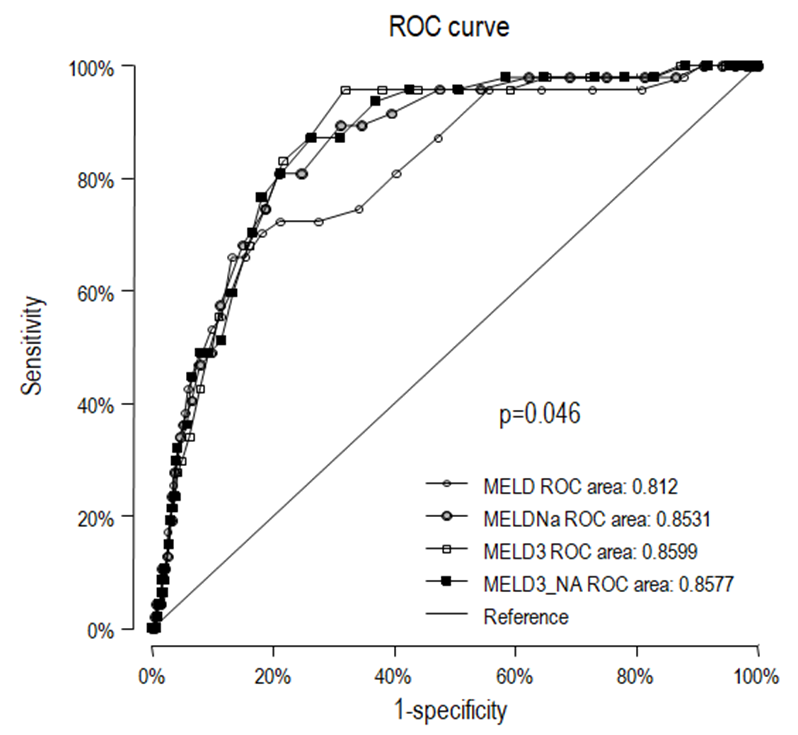


Supplementary Figure 2. Comparison of Areas Under the Curve for 90 day mortality prediction on the Waiting List between the different scoring systems. MELD3.0 offered the best prediction (p=0.046). Compared to MELD, MELD3.0 offered a better prediction (p=0.026), but was not statistically better than MELDNa (p=0.36). Transplanted patients in the first 90 days included.
