# Supplementary material for: Validation of MELD3.0 in 2 centers from different continents
Source: Hepatol Commun. 2024 Jul 31;8(8):e0504. doi: 10.1097/HC9.0000000000000504 (PMC12333758; doi:10.1097/HC9.0000000000000504)
Supplement: SUPPLEMENTARY MATERIAL [file hc9-8-e0504-s003.docx]

**Supplementary table 1. Baseline characteristics by transplant status at 90 days.**

| **Variable** | **Not transplanted**  **(n=436)** | **Transplanted**  **(n=182)** | **p** |
| --- | --- | --- | --- |
| Sex (male) | 260 (59.6%) | 117 (64.3%) | 0.320 |
| Age(years) | 55.72 ± 10.35 | 55.38 ± 9.80 | 0.707 |
| Height (cm) | 169.00 ± 9.90 | 168.90 ± 8.48 | 0.875 |
| Weight (kg) | 78.82 ± 20.79 | 78.46 ± 17.92 | 0.835 |
| MELD^¥^ | 16.56 ± 5.87 | 21.47 ± 7.88 | **<0.001** |
| MELDNa^¥^ | 18.34 ± 6.15 | 23.25 ± 7.71 | **<0.001** |
| MELD3.0 ^¥^ | 18.79 ± 6.27 | 23.77 ± 8.13 | **<0.001** |
| MELD3.0 no albumin ^¥^ | 18.79 ± 6.34 | 24.08 ± 8.35 | **<0.001** |
| Aetiology   - ALD - Viral - MASLD - AIH - PSC - PBC - Others | \| 165 (37.8%) \| \| --- \| \| 101 (23.1%) \| \| 90 (20.6%) \| \| 27 (6.2%) \| \| 54 (12.4%) \| \| 36 (8.3%) \| \| 65 (14.9%) \| | \| 89 (48.9%) \| \| --- \| \| 38 (20.9%) \| \| 32 (17.6%) \| \| 11 (6.0%) \| \| 19 (10.4%) \| \| 16 (8.8%) \| \| 17 (9.4%) \| | **0.012**  0.155  0.438  1.000  0.585  0.874  0.069 |

Results of quantitative variables expressed as Mean ± SD. Categorical variables are expressed as n (%).Welch Two Sample t-test for comparison between both cohorts (quantitative variables).Pearson's Chi-squared or Fisher’s exact test (categorical variables). ^¥^ At the time of listing. ALD: alcohol-related liver disease. MASLD: metabolic dysfunction-associated steatotic liver disease. AIH: autoimmune hepatitis. PSC: primary sclerosing cholangitis. PBC: primary biliary cholangitis.
