# Supplementary material for: Validation of MELD3.0 in 2 centers from different continents
Source: Hepatol Commun. 2024 Jul 31;8(8):e0504. doi: 10.1097/HC9.0000000000000504 (PMC12333758; doi:10.1097/HC9.0000000000000504)
Supplement: SUPPLEMENTARY MATERIAL [file hc9-8-e0504-s004.docx]

**Supplementary table 2.**Multivariate Cox regression model associated with mortality.

| **Variable** | **AdjustedHR (95%CI)** | **p** |
| --- | --- | --- |
| Not receiving a transplant | 4.760 (2.857 - 7.932) | **<0.001** |
| Toronto Cohort | 1.024 (0.652 - 1.609) | 0.917 |
| Sex male | 1.024 (0.734 - 1.429) | 0.887 |
| Age (years) | 1.048 (1.028 - 1.067) | **<0.001** |
| Baseline MELD3.0 ≥ 30 | 4.530 (2.754 - 7.452) | **<0.001** |
| PVT | 0.655 (0.391 - 1.097) | 0.108 |

Transplant is considered a time-dependant variable. HR: Hazard Ratio witha 95% confidence interval. PVT: Portal vein thrombosis
